# Cerebral small vessel disease and intracranial bleeding risk: Prognostic and practical significance

**DOI:** 10.1177/17474930221106014

**Published:** 2022-06-24

**Authors:** Jonathan G Best, Aaron Jesuthasan, David J Werring

**Affiliations:** Stroke Research Centre, UCL Queen Square Institute of Neurology, University College London, London, UK

**Keywords:** Brain microbleeds, intracerebral hemorrhage, MRI, prevention, cerebral hemorrhage, hemorrhage

## Abstract

Balancing the risks of recurrent ischemia and antithrombotic-associated bleeding, particularly intracranial hemorrhage (ICH), is a key challenge in the secondary prevention of ischemic stroke and transient ischemic attack. In hyperacute ischemic stroke, the use of acute reperfusion therapies is determined by the balance of anticipated benefit and the risk of ICH. Cerebral small vessel disease (CSVD) causes most spontaneous ICH. Here, we review the evidence linking neuroimaging markers of CSVD to antithrombotic and thrombolytic-associated ICH, with emphasis on cerebral microbleeds (CMB). We discuss their role in the prediction of ICH, and practical implications for clinical decision making. Although current observational data suggest CMB presence should not preclude antithrombotic therapy in patients with ischemic stroke or TIA, they are useful for improving ICH risk prediction with potential relevance for determining the optimal secondary prevention strategy, including the use of left atrial appendage occlusion. Following ICH, recommencing antiplatelets is probably safe in most patients, while the inconclusive results of recent randomized controlled trials of anticoagulant use makes recruitment to ongoing trials (including those testing left atrial appendage occlusion) in this area a high priority. Concern regarding CSVD and ICH risk after hyperacute stroke treatment appears to be unjustified in most patients, though some uncertainty remains regarding patients with very high CMB burden and other risk factors for ICH. We encourage careful phenotyping for underlying CSVD in future trials, with the potential to enhance precision medicine in stroke.

## Introduction

Antithrombotic and thrombolytic medications are central to stroke medicine, but inevitably risk bleeding, most seriously, intracranial hemorrhage (ICH). Cerebral small vessel disease (CSVD) is present in most patients with antithrombotic-associated ICH,^1^ and can be readily assessed in patients with suspected stroke given the near-universal use of computed tomography (CT), widespread availability of magnetic resonance imaging (MRI), and consensus definitions and visual rating scales for neuroimaging markers of CSVD (Table 1).^2^ CSVD markers appear prognostically significant, but it remains unclear whether they should influence treatment decisions. Here, we review the association of CSVD with ICH and its clinical implications in patients taking antithrombotics for secondary prevention after ischemic stroke or transient ischemic attack (TIA), including those with previous ICH and cerebral amyloid angiopathy (CAA), and in patients receiving hyperacute treatment for ischemic stroke.

**Table 1.** MRI markers of CSVD.

Marker	Definition	Pathophysiology	Example
Cerebral microbleed	Small, rounded areas (diameter ⩽ 10 mm) of signal void. Best seen on SWI or T2*weighted GRE as decreased signal and blooming effect.	Hemosiderin-laden macrophages at the site of previous extravasation of blood from small cerebral arteries. Other mechanisms might include microaneurysms and hemorrhagic transformation of microinfarcts.	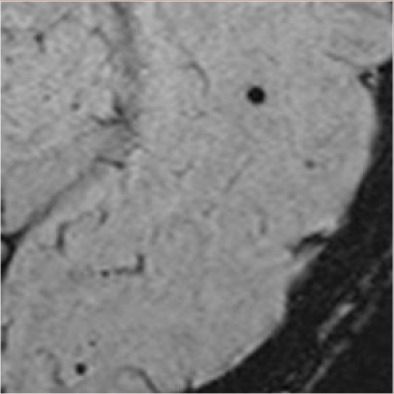
White matter hyperintensity	Increased signal on T2-weighted and FLAIR MRI. Mainly located in white matter; subcortical hyperintensities in deep gray matter and brainstem are not included in WMHs.	Gliosis, demyelination, blood brain barrier impairment. Associated with abnormalities of small cerebral arteries.	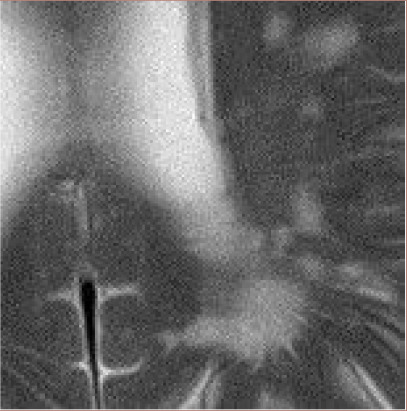
Lacune	Round or ovoid fluid filled cavity (3-15 mm diameter in the axial plane) mostly in subcortical regions; hyperintensity on T2-weighted MRI; decreased signal in FLAIR (often with hyperintense halo) and T1-weighted images. Signal characteristics similar to CSF.	Usually due to infarction in the territory of a single perforating artery, but can also result from small hemorrhages or occlusion of a larger artery (e.g., striatocapsular infarct).	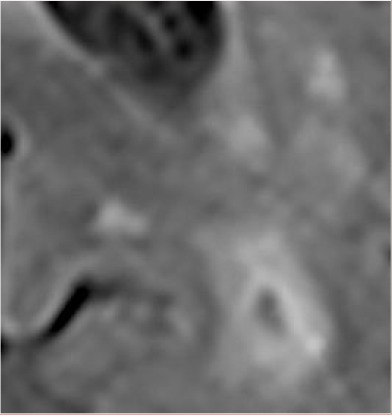
Enlarged perivascular spaces	Fluid-filled spaces that follow the typical course of a vessel through gray or white matter. Found in the basal ganglia or centrum semiovale. Similar signal intensity with CSF; increased T2-weighted signal decreased signal on FLAIR and T1-weighted MRI.	Enlargement of the anatomical compartment between vessel walls and the glia limitans of the blood–brain barrier; may reflect impaired interstitial fluid drainage from the brain associated with structural and functional consequences of CSVD. When located in the basal ganglia, EPVS are associated with arteriolosclerosis. EPVS in the centrum semiovale appear to be related to CAA.	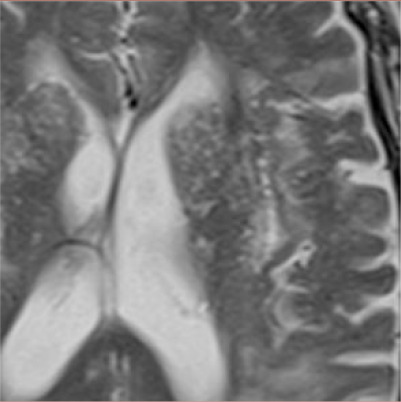

## Cerebral small vessel disease and antithrombotic therapy after ischemic stroke or TIA

In patients with ischemic stroke, antiplatelet monotherapy reduces long-term recurrence by around one-quarter but increases ICH risk by one-fifth. As the absolute risk of recurrent ischemic stroke is substantially higher, antiplatelets reduce stroke risk by one-fifth overall.^3^ Antiplatelets are similarly effective after ischemic stroke attributed to small vessel occlusion, based on the AICLA, CSPS, and CATS trials.^4^ In patients with atrial fibrillation (AF), oral anticoagulation with a vitamin K antagonist (VKA) reduces overall stroke risk by two-thirds, despite increasing ICH risk twofold.^5^ Direct oral anticoagulants (DOACs) are similarly effective, but pose around half the ICH risk of VKAs.^6^ Antithrombotic therapy therefore clearly benefits ischemic stroke patients overall, but the expected individual net benefit varies according to the baseline risks of recurrence and ICH. Whether neuroimaging features of CSVD might identify patients at high risk of ICH—and net harm from antithrombotic therapy—has attracted considerable interest.

### Cerebral microbleeds

**Figure 1.**
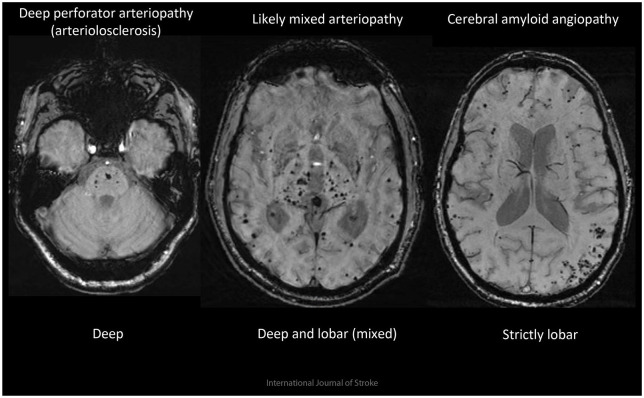
Examples of different underlying cerebral microbleed disease patterns associated with ICH.

CMBs have many causes (Table 2), but the commonest is CSVD. Deep CMBs are associated with arteriolosclerosis, while multiple strictly lobar CMBs have good sensitivity and specificity for pathologically-confirmed CAA, at least in patients with ICH.^7,8^ A mixed CMB distribution can indicate severe diffuse arteriolosclerosis, mixed arterioloscleriosis and CAA, or some rare small vasculopathies, including monogenetic diseases (Figure 1).

**Table 2.** Some conditions associated with cerebral microbleeds.

Cerebral small vessel diseases	Other brain disease	Systemic diseases
• Arteriolosclerosis • Sporadic cerebral amyloid angiopathy (CAA) • Inflammatory CAA • Iatrogenic CAA • Alzheimer’s disease (with CAA) • Genetic small vessel diseases • CADASIL • CARASIL • CARASAL • COL41A • Hereditary CAA • Fabry disease	• Moya–Moya syndrome • CNS vasculitis • Radiation vasculopathy • Reversible Cerebral Vasoconstriction Syndrome • (Multiple sclerosis)	• Infective endocarditis • Cardiac valve replacement / cardiopulmonary bypass • Antiphospholipid syndrome • Sneddon syndrome • Sickle disease • Thrombotic thrombocytopaenic purpura • Hemophilias • Critical care encephalopathy • COVID-19 • ECMO • Chronic renal impairment

**Figure 2.**
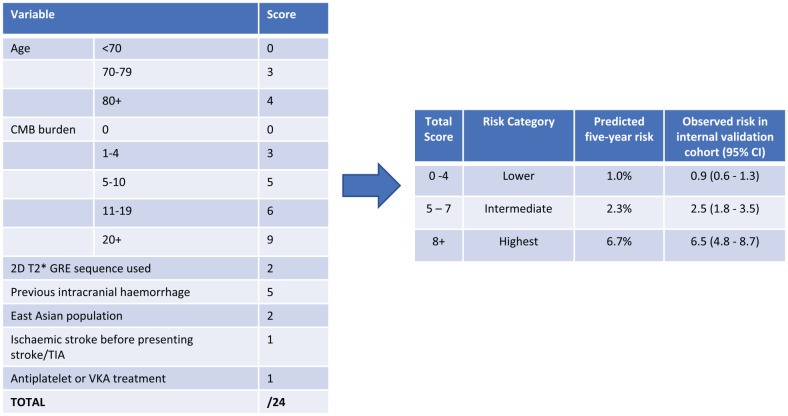
MICON-ICH risk score.

Numerous observational studies link CMBs to ICH risk. In patients prescribed oral anticoagulants after cardioembolic ischemic stroke or TIA in the CROMIS-2 and HERO studies, CMB presence was independently associated with a roughly threefold increase in symptomatic ICH risk.^9,10^ A pooled analysis of two large cohorts found a similar association in patients with ischemic stroke of any cause, mainly prescribed antiplatelet monotherapy.^11^ A subsequent pooled analysis of 38 studies comprising 20,322 patients prescribed antithrombotics after ischemic stroke or TIA found rapidly increasing ICH risk with CMB burden,^12^ and showed that a CMB-based model (MICON-ICH, Figure 2) outperformed existing bleeding risk models in ICH prediction.^13^ However, CMBs were also associated with ischemic stroke, with absolute incidence exceeding that of ICH regardless of CMB burden and distribution.^12^ Thus, current observational evidence suggests that CMBs should not preclude standard antithrombotic therapy after ischemic stroke or TIA.

However, there remain no dedicated randomized controlled trials (RCTs) to show this definitively. A secondary analysis of the NAVIGATE-ESUS trial, which compared rivaroxaban with aspirin in patients with presumed embolic stroke, found no interaction between CMB presence and antithrombotic treatment type regarding ICH, recurrent stroke, or death.^14^ In the SPS3 trial of dual antiplatelet therapy versus aspirin monotherapy in patients with lacunar infarction, no interaction between CMB presence and treatment allocation was found for recurrent stroke (including ICH).^15^ These studies support antithrombotic use in patients with CMBs, although they included very few patients with 11+ CMBs. CMBs might nevertheless influence secondary prevention, by identifying patients at whom to target measures to reduce bleeding risk, including close follow-up, intensive hypertension treatment, and addressing modifiable risk factors such as alcohol intake.

Left atrial appendage occlusion (LAAO) is intended to reduce the risk of ischemic stroke in patients with AF without requiring long-term anticoagulation. Although its efficacy compared with OAC remains controversial, it offers a lower risk of bleeding,^16^ possibly even compared with DOACs if discounting periprocedural events,^17^ and theoretically might be most indicated in patients at highest ICH risk. This is being investigated in patients with symptomatic ICH in the STROKECLOSE (NCT:02830152) and A3ICH (NCT:03243175) trials. Whether patients with higher CMB burdens without previous symptomatic ICH might also benefit from LAAO compared with OAC should be investigated. Finally, the optimal antithrombotic might differ depending on CMB presence. In a secondary analysis of the PICASSO trial, which randomized patients at high bleeding risk (defined by previous ICH or presence of multiple CMBs) to cilostazol or aspirin, patients with CMBs, but not ICH, had a lower risk with cilostazol of ICH (HR 0.08, 95% CI = 0.01–0.60) and (nonsignificantly) major vascular events (HR 0.64, 95% CI = 0.41–1.01).^18^

### Other neuroimaging markers of CSVD

Based on a comprehensive meta-analysis, the risk of incident ICH increases with the presence of extensive white matter hyperintensities (WMH) (HR 3.17, 95% CI = 1.54–6.52) and chronic brain infarcts (HR 3.81, 95% CI = 1.75–8.27) on MRI.^19^ The relationship between enlarged perivascular spaces (EPVS) in the basal ganglia and ICH is less clear.^11,20,21^ Despite their probable association with CAA in ICH patients,^22^ EPVS in the centrum semiovale are not associated with ICH in ischemic stroke patients. Whether these markers could improve ICH prediction is uncertain: markers of CSVD are correlated, and neither the inclusion of WMH burden nor lacunes improved ICH prediction over CMBs in the MICON-ICH prediction model development study.^13^ These markers are also associated with ischemic stroke risk, so are unlikely to identify patients with an ICH risk exceeding that of ischemic stroke.^19,23^

## Antithrombotic therapy in patients with previous ICH or CAA

About one in three ICH patients take antithrombotic agents to prevent vaso-occlusive events, including ischemic stroke. Traditionally, antithrombotic drugs have been avoided in ICH survivors. However, it is now realized that there is not only a substantial risk of recurrence, but also an often similarly high risk of further cerebral and extracerebral ischemia, particularly in those with non-lobar ICH or AF.^24^ As observational data using the modified Boston diagnostic criteria for CAA indicate that the baseline risk of ICH recurrence is much higher in patients with probable CAA than those without,^25^ the burden of CSVD might be an important consideration in this high risk group.

### Antiplatelets

RESTART,^26^ a UK multicentre open label pilot phase RCT, included 537 participants at a median time of 76 days after antithrombotic-associated ICH. Over a median follow-up of 2 years (IQR 1–3) 4% of those allocated to antiplatelet therapy had recurrent ICH compared with 9% of those allocated to avoid antiplatelet therapy (aHR 0.51, 95% CI = 0.25–1.03), with no difference in major occlusive vascular events, but a reduction in all serious vascular events as defined by the Antithrombotic Trialists’ Collaboration. However, during extended follow-up to 5 years,^27^ antiplatelet therapy did not reduce ICH recurrence or major vascular events. An imaging sub-study (*n* = 254 with MRI) found no evidence of more frequent recurrent ICH in patients with 2+ CMBs versus one or no CMBs, nor in subgroups according to CMB burden or strictly lobar location (suggesting CAA).^28^ RESTART therefore gives some reassurance that restarting antiplatelet therapy after ICH may be safe with regard to recurrent ICH, with no signal that the burden or type of underlying CSVD modifies this result. However, there remain concerns about the small sample size and selection bias (average ICH volume 4 mL, smaller than many ICH seen in clinical practice). Moreover, RESTART included few participants with probable CAA, the subgroup of highest concern for ICH risk.^28^ Thus, further RCTs including subgroups related to CSVD presence, type, or severity are needed to validate these findings.

### Oral anticoagulants

Rather surprisingly, observational studies suggest that resuming anticoagulation following ICH is not associated with a higher risk of recurrent ICH,^29^ even for those with lobar ICH or imaging evidence of CAA.^30^ These results might have been confounded by indication and treatment bias; moreover, most patients on anticoagulation in these older studies were treated with VKAs. RCT data are therefore urgently needed.

Two small trials (SoSTART and APACHE AF) have recently published data. SoSTART randomized 203 participants with previous spontaneous ICH (92% intracerebral, 84% OAC-associated) to start or avoid OAC at a median time from ICH onset of 115 days (IQR 49–265).^31^ Over a median follow-up of 1.2 years, those allocated to OAC (almost all a NOAC) had double the rate of ICH recurrence (8.0%) than those avoiding OAC (8.0% vs. 3.9%, *p* = 0.15), but roughly half the risk of any stroke (11% vs. 22%, *p* = 0.08) and any stroke or vascular death (12% vs. 23%, *p* = 0.09). In APACHE-AF, 101 participants with ICH in the previous 90 days (median time 46 days from onset) received apixaban or no anticoagulation.^32^ Over a median follow-up of 1.9 years, the primary outcome of stroke or vascular death occurred in 26% of those on apixaban and in 24% of those avoiding anticoagulation (aHR 1.05, 95% CI = 0.48–2.31). More participants in the apixaban group experienced recurrent ICH (aHR 4.08, 95% CI = 0.45–39.91), but there was no difference between groups in the rate of ischemic stroke.

These inconclusive results underline the need for more RCTs to identify subgroups of patients with AF and ICH in whom the effect of restarting anticoagulation might be either beneficial or hazardous. Careful phenotyping for underlying CSVD presence, type and burden might identify such groups. Recruitment to relevant ongoing trials—including ASPIRE (NCT:03907046), ENRICH-AF (NCT:03950076), PRESTIGE-AF (NCT:03996772) and STATICH (NCT: 03186729)—is encouraged.

## Cerebral small vessel disease and hyperacute treatment of ischemic stroke

Concern regarding acute reperfusion therapies and CSVD predates their use for ischemic stroke: in the TIMI-II trial, over half of patients suffering a fatal ICH after treatment with recombinant tissue plasminogen activator (tPA) for acute myocardial infarction had CAA at post-mortem.^33^ Subsequently, in patients receiving intravenous thrombolysis (IVT) for acute ischemic stroke in the NINDS-tPA trial, one-fifth of symptomatic ICH occurred outside of the acute infarct,^34^ further implicating CSVD in post-thrombolysis ICH.

### CMBs and IVT

CMBs are found on pretreatment MRI in around 20% of patients with acute ischemic stroke who subsequently undergo IVT.^35,36^ In a meta-analysis including 2028 patients, 8.5% of participants with at least one CMB on pretreatment MRI developed symptomatic ICH during follow-up, compared with 3.9% of those without (adjusted odds ratio (aOR) 2.26, 95% CI =1.49–3.49).^35^ In another meta-analysis including 1808 patients,^36^ symptomatic ICH risk appeared markedly elevated in patients with 11+ CMBs (pooled incidence 46.9%, 95% CI = 22.8–72.5), compared with patients without CMBs (3.2%, 95% CI = 1.7–6.1) or with 1–10 CMBs (6.4%, 95% CI = 4.2–9.5). However, such patients were rare (*n* = 15). A pooled analysis of individual patient data from eight studies (seven included in previous meta-analyses) associated the presence of 11+ CMBs with symptomatic ICH (OR 3.65, 95% CI = 1.17–11.42), though not with CMB presence overall (aOR 1.42, 95% CI = 0.86–2.35).^37^ Informed by these studies, the European Stroke Organisation makes a weak recommendation against IVT in patients with 11 or more CMBs,^38^ although the American Stroke Association does not.^39^ This recommendation should be considered alongside a modeling study that estimated the relative treatment effect of IVT on functional outcome in patients with 11+ CMBs using a multistep algorithm.^40^ It concluded that IVT might cause net harm only in a subset of these patients, mainly those over 80 years with severe stroke or longer onset-to-treatment times.

A subgroup analysis of the WAKE-UP RCT supports IVT use in patients with CMBs. In 429 patients receiving MRI-guided IVT for ischemic stroke of unknown onset, the benefit of IVT regarding functional outcome was similar in patients with and without CMBs (OR for mRS 0–1 at 90 days with IVT: CMBs present: 2.6, 95% CI = 0.7–9.6; CMBs absent: 1.71, 95% CI = 1.08–2.70; interaction *p* = 0.55).^41^ Although both IVT and CMB presence increased symptomatic ICH risk, there was no interaction between the two (*p* = 0.52). The proportion of patients with an excellent functional outcome did not change with increasing CMB burden, although only 15/429 (3.5%) patients had 5+ CMBs. Much larger studies would be needed to establish definitively whether patients with 11+ CMBs should receive IVT, but are unlikely to be feasible. Moreover, delaying IVT by more than 10 min to obtain screening MRI might lead to net harm.^40^

In a pooled analysis of individual patient data, the associations between lobar and deep CMBs and symptomatic ICH after IVT were similar, whether considering CMB presence or burden.^37^ This might reflect the weaker association between lobar CMBs and CAA in patients without previous ICH, and concords with observational data showing no association between lobar CMB distribution and long-term ICH risk in ischemic stroke patients taking antithrombotics.^12^ Patients with previous symptomatic ICH are generally not considered for thrombolysis in the absence of a definitively treated cause, regardless of CMB burden or distribution.^38^

### CMBs and mechanical thrombectomy

In a retrospective cohort study of 513 patients treated with stent retriever or aspiration devices, CMB presence or burden on pretreatment MRI was associated neither with symptomatic ICH, nor reperfusion probability or functional outcome in adjusted analyses.^42^ A smaller previous observational study reached similar conclusions.^43^ Interestingly, a recent retrospective analysis of the German Stroke Registry suggests that patients meeting modified Boston criteria for CAA might also benefit from MT (OR for mRS 0–2 with successful reperfusion: 6.82, 95% CI = 1.77–26.3), with similar rates of post-MT ICH to patients without CAA. However, only 10/28 patients met criteria for “probable” rather than “possible” CAA, and patients were almost exclusively diagnosed on the basis of multiple CMBs or cortical superficial siderosis (cSS), rather than previous symptomatic ICH, making this a lower-risk population than unselected CAA patients.^44^

### CT markers

Observational studies consistently link leukoaraiosis to symptomatic ICH and poor functional outcome in patients receiving IVT.^45^ However, in a subgroup analysis of the NINDS-tPA trial, severe leukoaraiosis was associated with a nonsignificant increase in the risk of symptomatic ICH (14.3% vs. 6.3%) in tPA-treated patients, but did not modify the effect of IVT within 3 h of stroke onset on functional outcome.^46^ In the IST-3 trial, which included many participants over the age of 80 years and permitted IVT up to 6 h from onset, severe leukoaraiosis was not significantly associated with sICH (aOR 1.15, 0.77–1.70), and no interaction with treatment allocation was found for functional outcome or symptomatic ICH.^47^ Regarding MT, a secondary analysis of the MR CLEAN trial found no difference in treatment effect between patients with severe leukoaraiosis (aOR 1.95, 95% CI = 0.90–4.20) and those without (aOR 1.93, 95% CI = 1.31–2.82).^48^ The impact of MRI-defined WMH burden appears to be similar.^49^

Old infarcts on CT (including lacunes) also do not modify the benefit of IVT. In IST-3, old infarcts predicted symptomatic ICH (OR 1.72, 95% CI = 1.18–2.51) and worse functional outcome (OR for OHS 0–1: 0.79, 95% CI = 0.64–0.96) but did not interact with treatment allocation. In the dose arm of the ENCHANTED trial, which compared low-dose with standard dose alteplase in 3310 participants (younger than in IST-3), old infarcts were associated with worse functional outcome (OR for mRS 0–2 at 3 months: 0.78, 95% CI = 0.64–0.94), regardless of dose, but not symptomatic ICH.^50^

### Acute lacunar infarction

Small artery occlusion might occur by non-thrombotic mechanisms, leading to concerns about IVT use in acute lacunar infarction. However, a recent meta-analysis of all available comparative observational and randomized data, including subgroup analyses of the IST-3, NINDS, and WAKE-UP trials, found that IVT was associated with better odds of excellent functional outcome (OR 1.53, 95% CI = 1.29–1.82).^51^ As many studies defined small vessel infarction using clinical classification schemes rather than imaging, the findings from the WAKE-UP trial,^52^ which used diffusion-weighted MRI, are particularly important, with no heterogeneity in the treatment effect of alteplase between patients with lacunar and non-lacunar infarction (*p* = 0.94). Withholding IVT therefore appears unjustified.

## Conclusion

**Figure 3.**
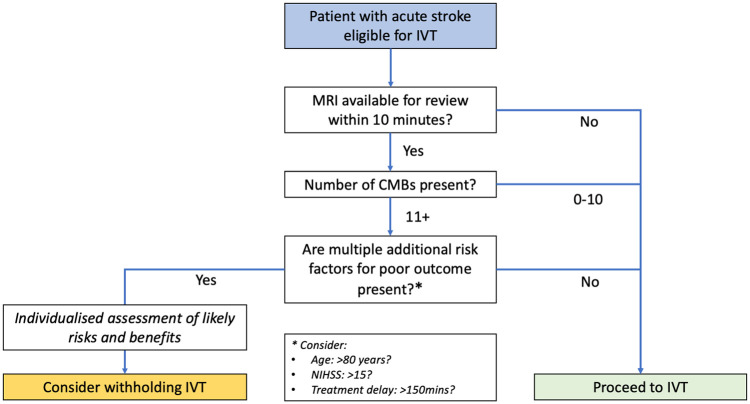
Flowchart for hyperacute ischemic stroke treatment in presence of CMBs.

CMBs are associated with ICH risk and improve its prediction. Their presence should generally not affect antithrombotic use for secondary stroke prevention, although whether they could identify patients who might benefit from novel antithrombotic regimens or non-pharmacological approaches remains to be determined. In the hyperacute setting, patients with CSVD benefit similarly from IVT and MT to those without, though have worse functional outcomes in general, and, in the case of IVT, might have higher rates of symptomatic ICH. Uncertainty exists regarding patients with 11+ CMBs, and an individualized assessment of the likely risks and benefits seems appropriate in these patients. When MRI is already available and can be quickly interpreted, reviewing this before IVT would be reasonable (Figure 3), but treatment should not be delayed to obtain MRI.
